# ROCK Inhibitor Is Not Required for Embryoid Body Formation from Singularized Human Embryonic Stem Cells

**DOI:** 10.1371/journal.pone.0100742

**Published:** 2014-11-03

**Authors:** Giuseppe Pettinato, Wendy S. Vanden Berg-Foels, Ning Zhang, Xuejun Wen

**Affiliations:** 1 Department of Chemical and Life Science Engineering, Virginia Commonwealth University, Richmond, Virginia, United States of America; 2 Department of Biomedical Engineering, Virginia Commonwealth University, Richmond, Virginia, United States of America; 3 Department of Bioengineering, Clemson University, Clemson, South Carolina, United States of America; 4 Department of Craniofacial Biology, Medical University of South Carolina, Charleston, South Carolina, United States of America; 5 Institute for Biomedical Engineering and Nano Science (iNANO), Shanghai East Hospital, Tongji Medical School, Tongji University, Shanghai, People's Republic of China; Instituto Butantan, Brazil

## Abstract

We report a technology to form human embryoid bodies (hEBs) from singularized human embryonic stem cells (hESCs) without the use of the p160 rho-associated coiled-coil kinase inhibitor (ROCKi) or centrifugation (spin). hEB formation was tested under four conditions: +ROCKi/+spin, +ROCKi/-spin, -ROCKi/+spin, and -ROCKi/-spin. Cell suspensions of BG01V/hOG and H9 hESC lines were pipetted into non-adherent hydrogel substrates containing defined microwell arrays. hEBs of consistent size and spherical geometry can be formed in each of the four conditions, including the -ROCKi/-spin condition. The hEBs formed under the -ROCKi/-spin condition differentiated to develop the three embryonic germ layers and tissues derived from each of the germ layers. This simplified hEB production technique offers homogeneity in hEB size and shape to support synchronous differentiation, elimination of the ROCKi xeno-factor and rate-limiting centrifugation treatment, and low-cost scalability, which will directly support automated, large-scale production of hEBs and hESC-derived cells needed for clinical, research, or therapeutic applications.

## Introduction

Human embryonic stem cells (hESCs) are pluripotent, with the ability to differentiate into all somatic and germ cell types in the body. As a result, hESCs have widespread implications for human developmental biology and cell biology, drug discovery, and transplantation medicine for human tissue regeneration [1], [2]. Protocols have been developed to induce differentiation of hESCs into a wide variety of cell types, including hematopoietic cells [3], [4], cardiomyocytes [5], [6], neural progenitors and functional neurons [7]–[9], hepatocytes [10], [11], and pancreatic beta cells [12], [13], among others. A significant challenge for the clinical translation of hESC research successes *in vitro* and in animal models is the efficient production of a sufficient number of differentiated cells needed for patient treatment. Key requirements for clinical translation include the delivery of a homogeneous, functional cell population [14], defined xeno-free culture conditions [9], and easy scale-up with automation technology to meet demand in a cost-effective manner [15].

Formation of an embryoid body (hEB) is the first step in hESC differentiation protocols [16], [17]. In three-dimensional aggregates, hESCs form cell-cell contacts, spontaneously differentiate to form the three embryonic germ layers of endoderm, mesoderm, and ectoterm, and recapitulate features of pregastulation and early gastrulation [16], [18]. Because hESCs have low survival rates as dissociated single cells [19], hEBs have typically been formed using hESC colonies or colony pieces that are cultured in suspension [16], [20] or in hanging drops [17], [21] to promote aggregation. However, thus-derived hEBs have both pre-existing and newly formed cell-cell contacts, and exhibit a broad size distribution and irregular geometries, both of which are associated with asynchronous differentiation [15], and reduced homogeneity and reproducibility of the resulting cell population [22], [23].

More recent approaches to hEB formation have used dissociated single-cell suspension of hESCs as the input population. Treatment with the p160 Rho-associated coiled-coil kinase (ROCK) inhibitor (ROCKi, Y-27632) has been widely used to promote survival of dissociated hESCs after passages [19] and assist EB formation from dissociated single-cell suspension of hESCs [15], [24]. The exact mechanism by which ROCKi promotes hESC survival and hEB formation is unknown; yet, evidence suggests that ROCKi may prevent anoikis associated with loss of cell-cell contacts [25], [26]. Nonetheless, ROCKi is a xeno-factor with little known about its potential downstream effects. ROCKi has been shown to bias cell fate toward residual pluripotency in neural differentiation studies, making these cells unsuitable for cell therapies [8]. In addition to heavy dependence of hEB formation on the presence of ROCKi, most protocols have applied centrifugation as a means to force cell aggregation [27], [28]. Although centrifugation may avoid exposure of hESCs to the ROCKi xeno-factor, it is not conducive to high throughput, automated production of hEBs.

When compared to cell colonies/clumps, dissociated single cell suspension represents a more uniform inputting population that makes robotic time-efficient large-scale production of hEBs possible to meet the demand of real-world applications. To form hEBs in large quantities from dissociated single-cell suspension of hESCs, researchers have recently turned to molds or plates that contain an array of microwells [15], [27]–[29]. To date, microwell-based hEB formation from dissociated hESCs in other labs has indicated no success in the absence of ROCKi or centrifugation [15], [27]–[29], likely due at least in part to the lack of efficient cell aggregation and control of cell-cell signaling and colony characteristics that are crucial for hESC survival, growth, and differentiation.

Here, we report a technology to form hEBs from singularized hESCs without the use of ROCKi or centrifugation. hEB formation was tested under four conditions: +ROCKi/+spin, +ROCKi/-spin, -ROCKi/+spin, and -ROCKi/-spin. Dissociated single cell suspension of hESCs was pipetted into non-adherent hydrogel molds containing defined micro-well arrays. For both tested hESC lines, i.e., BG01V/hOG (Invitrogen), and feeder-free H9 (WiCell Research Institute), hEBs of consistent size and spherical geometry were formed in each of the four conditions, including the -ROCKi/-spin condition. The hEBs formed without ROCKi and spin differentiated to develop the three embryonic germ layers and tissues derived from each of the germ layers. This simplified hEB production technology offers homogeneity in hEB size and shape to support synchronous differentiation, elimination of the ROCKi xeno-factor and rate-limiting centrifugation treatment, and low-cost scalability, which will directly support automated, large-scale production of hESC-derived cells needed for clinical, research, or therapeutic applications.

In addition to the technical advances pertinent to stem cell therapeutics, this study will gain insight into human embryogenesis especially cellular differentiation hierarchy, of which our knowledge is very rudimentary. Assess to various stages of human embryonic development would not be feasible due to both practical and ethical reasons, yet, model systems that allow direct observation of human developmental process are currently not available. hEBs address this critical need by offering a model system to study human development as well as a source of differentiated cells for scientific and clinical interest. Controlled reproducible high throughput production of uniform-sized hEBs as achieved using this approach represents a platform to recapitulate aspects of human developmental processes in vitro in which interplays among endogenous and exogenous factors during embryogenesis, hEB-mediated differentiation, and tissue formation are essential. Further, successful generation of homogenous hEBs using our method that is free of ROCKi treatment or centrifugation has for the first time questioned the conventional concept regarding the necessity of ROCKi for hESC survival in single-cell suspension and hEB formation. Elucidating the key environmental factors to hESC survival, proliferation, and directed synchronous differentiation, as well as administering these factors using an engineering approach lays the foundations toward manipulating output cell populations for research, clinical, and industrial purposes.

## Materials and Methods

### hESC maintenance culture

BG01V/hOG (Invitrogen – R7799-105) and feeder-free H9 (WiCell Research Institute – Lot WB007–WA09) hESC lines were cultured according to supplier protocols. BG01V/hOG hESCs were expanded on a feeder layer of mitomycin C-inactivated (10 µg/ml, Invitrogen) mouse embryonic fibroblasts (MEFs, Millipore) in hESC medium (DMEM/F12 with 2 mM GLUTAMAX (Invitrogen), 20% (v/v) knockout serum replacement (Invitrogen), 0.1 mM nonessential amino acids (Invitrogen), 55 µM β-mercaptoethanol (Gibco), 4 ng/ml recombinant human bFGF (Inivitrogen), and 50 µg/ml hygromycin B (Invitrogen)). hESC colonies were passaged by cutting them into uniform pieces (StemPro EZPassage tool, Invitrogen). Following initial expansion, BG01V/hOG hESC were switched to matrigel (Growth Factor Reduced Matrigel, BD Bioscences) and expanded in chemically defined mTeSR1 medium (mTeSR1 Basal Medium with mTeSR1 5X supplement, Stem Cell Technologies). BG01V/hOG hESC were passaged five times to eliminate MEF from the culture. H9 hESC are cultured under a feeder-free condition. Their expansion protocol was the same as that for the feeder-free culture of the BG01V/hOG hESC. H9 colonies were passaged using 0.2 g/L Versene (EDTA) (Lonza). BG01V/hOG and H9 hESC colonies were incubated with Accutase (Innovative Cell Technologies) for 5 min at 37°C to form single cell suspensions.

### Fabrication of hydrogel microwells using stamps with micronipple arrays, and hEB formation

The stamp with micronipple arrays (**Fig. S1**) were first designed with a computer-aided design program (SolidWorks) to generate STereoLithography (STL) file, which was transferred to computer numerical controlled (CNC) ultra-high precision lathe (Siemens) to fabricate the micronipples at the Teflon stamp surface. Each micronipple was highly polished. Agarose (Sigma Aldrich) solution (2% w/w) was prepared in phosphate buffered saline (PBS) by heating to 50°C and pipetted into the culturewares, such as multiwall plates, and culture dishes. Then the stamp with micronipple arrays was pressed into the Agarose solution at room temperature for 2 minutes. High-quality uniform-sized microwells were imprinted in the Agarose hydrogels. The size of microwells can be well controlled through the use of stamps of different sized micronipples. In this study, the size of each microwell was 820 µm in diameter and 1300 µm in depth. Arrays were equilibrated with hEB culture medium (Iscove's Modified Dulbecco's Medium (1∶1 IMDM (Invitrogen) and F-12 Nutrient Mixture (Ham) (Invitrogen)), 5% fetal bovine serum (Gibco), 1% (v/v) insulin transferrin selenium-A supplement (Invitrogen), 55 µM monothioglycerol (Sigma Aldrich), 100 U/ml penicillin, and 0.1 mg/ml streptomycin (Gibco)) overnight at 37°C and 5% CO_2_.

For hEB formation, 50 µl of hESC suspension was pipetted into microwell arrays (**Fig. S1**). The suspension was allowed to sediment into the microwells for 10 min before additional medium was added. hEB formation was tested over the range of 10,000 to 35,000 hESCs per microwell in increments of 5,000 hESCs for each hESC cell lines.

Two treatments to promote hEB formation, the addition of ROCKi and centrifugation (spin), were examined as controls. In total, four treatment conditions were tested: +ROCKi/+spin, +ROCKi/-spin, -ROCKi/+spin, and -ROCKi/-spin. For the ROCKi treatment, 10 µM of ROCKi was added to the differentiation media. For the centrifugation treatment, the microwell arrays were centrifuged at 200 g for 5 min after cell suspension sedimentation.

### hEB viability

After 24-hr incubation in the microwell array, viabilities of hEBs and non-aggregated hESCs were evaluated by LIVE/DEAD staining (Catalog # L-7013, Molecular Probes) according to manufacturer instructions. Fluorescent images were acquired using a stereo fluorescent microscope (Eppendorf) and a confocal microscope (LSM 710, Carl Zeiss).

### hEB suspension culture and growth evaluation

After a 24-hr incubation in the microwell array, hEBs were placed in suspension culture and incubated at 37°C and 5% CO_2_ under gentle agitation. The differentiation medium was exchanged every two days. Images of the hEBs were acquired when the medium had been withdrawn from the culture dish. hEB sizes (mean ± S.D.) were quantified using Image Pro Plus (Media Cybernetics, v. 4.0) based upon the measurement of the cross sectional area of each hEB. hEB growth was monitored for 6 days in suspension culture.

### Transmission electron microscopy of cell-cell junctions

hEBs were fixed in 2% glutaraldehyde in 0.1 M cacodylate buffer for 1 hr, and rinsed with 0.1 M cacodylate buffered with 0.2 M sucrose. The hEBs were postfixed in 2% aqueous osmium tetroxide for 1 hr, rinsed with distilled water, dehydrated in a graded ethanol series, and embedded in resin (EMbed 812, Electron Microscopy Sciences). Seventy nanometer thick sections were supported on copper grids and double stained with uranyl acetate in methanol and with Reynolds lead citrate. Cell-cell junctions were imaged by transmission electron microscopy (JEOL 1010, JEOL).

### hEB differentiation

For cardiac differentiation, 0.14 µg/ml ascorbic acid (Sigma) was added to the differentiation medium. For neural differentiation, 0.1 µM retinoic acid (Sigma) was added to neural differentiation medium, which consisted of DMEM/F12 (Invitrogen), 1% (v/v) N2 supplement (Gibco), 0.2 µM ascorbic acid (Sigma), 10 ng/ml GDNF (PeproTech Inc.), 10 ng/ml BDNF (PeproTech Inc.), 5 ng/ml IGF-1 (PeproTech Inc.), 1 µM cAMP (Sigma), 2 µg/ml heparin (Sigma), and 1% (v/v) nonessential amino acids (Invitrogen). For pancreatic differentiation, 100 ng/ml activin A (PeproTech Inc.), 10 mM nicotinamide (Sigma), and 10 ng/ml EGF (Sigma) were added to the differentiation medium. Differentiation for cardiac, neural, and pancreatic differentiation was evaluated at 25, 26, and 18 days, respectively. Differentiation was performed in triplicate.

### Gene expression analysis

RT-PCR was performed to verify the presence of characteristic gene markers of differentiation. RNA was extracted using Trizol reagent (Invitrogen). RNA concentration and purity was measured by spectrophotometry (NanoDrop 2000, Thermo-Scientific). RNA was reverse transcribed to cDNA using the MMLV enzyme (Moloney Murine Leukemia Virus Reverse Transcriptase, Promega). One microgram of RNA was used per sample. cDNA was amplified using Taq polymerase (MBL). All procedures were conducted according to the manufacturer's instructions. Amplification was performed using the following parameters: one cycle of 94°C for 4 min, 30–35 cycles of denaturation at 94°C for 30 sec, and annealing at 60°C for 30 sec. The following genes were evaluated: For cardiac differentiation: alpha actin, GATA-4, GATA-6, Tbx-20, Tbx-5, Hand-2; For neural differentiation: Pax-6, Nestin, Beta-3, Tyr-Hyd, GABA; For pancreatic differentiation: Ngn3, Pdx-1, Insulin, PC-1/3, PC-2, GK, Sur-1, Kir-6.2, Glut-2, Isl-1, SOX17, Pax-6, and Nkx-6.1. GAPDH was used as the reference housekeeping gene.

### Immunofluorencence visualization of differentiation

For immunolocalization, hEBs and differentiation cultures were fixed with 4% (w/v) paraformaldehyde for 15 min, permeablized with 0.3% (v/v) Triton-X 100 in PBS for 10 min, and blocked with 0.5% (v/v) goat serum in PBS for 1 hr. Samples were incubated with the primary antibody at 4°C overnight and then with the secondary antibody at room temperature for 1 hr. Nuclei were stained with 4′,6-diamidino-2-phenylindole (DAPI) in PBS for 5 min. The following primary antibodies were used to stain the three hEB germ layers: mouse anti-alpha 1 fetoprotein (abcam, ab3980, 5 µg/ml), rabbit anti-SOX1 (abcam, ab22572, 4 µg/ml), and goat anti-brachyury (Santa Cruz, as-17743, 1∶50). The secondary antibodies were Cy2-AffiniPure goat anti-mouse IgG, Fc Subclass 1 Specific (Jackson ImmunoResearch, 1∶100), Cy3-AffiniPure goat anti-rabbit IgG (H+L) (Jackson ImmunoResearch, 1∶100), Cy5-conjugated AffiniPure rabbit anti-goat IgG (Jackson ImmunoResearch, 1∶100). The following primary antibodies were used for cardiac differentiation markers: mouse monoclonal anti-alpha cardiac actin (Sigma, A9357, 1∶100) and rabbit anti-cardiac troponin I–C (Santa Cruz, sc-98830, 1∶25). The secondary antibodies were Cy2-AffiniPure goat anti-mouse IgG, Fc Subclass 1 Specific (Jackson ImmunoResearch, 1∶100), Cy3-AffiniPure goat anti-rabbit IgG (H+L) (Jackson ImmunoResearch, 1∶100). The following primary antibodies were used for pancreatic differentiation markers: insulin (Sigma, I2018, 1∶100), C-peptide (Abcam, ab30477, 1∶100) and GLUT-2 (Abcam, ab54460, 1∶100). The secondary antibodies were Cy2-AffiniPure goat anti-mouse IgG, Fc Subclass 1 Specific (Jackson ImmunoResearch, 1∶100), Cy3-AffiniPure goat anti-rabbit IgG (H+L) (Jackson ImmunoResearch, 1∶100). Fluorescent images were acquired with a confocal microscope (Leica, SP5).

### Dithizone staining

A 0.01% dithizone (DTZ) (Merck) stock solution was prepared in dimethyl sulfoxide. hEBs in the pancreatic differentiation protocol were stained by adding DTZ stock solution to the culture medium at 0.01% (v/v), incubating the hEBs at 37°C for 15 minutes, and then rinsing thrice in HBSS.

### Western-blot analysis

Western blots were performed to detect proteins characteristic of cardiac differentiation. Whole proteins were extracted from the cells using radio-immunoprecipitation assay buffer (Thermo Scientific) according to manufacturer instructions. The protein content was quantified using the Bradford method. Absorbance at 595 nm was read using a microplate reader (Spectra max 384 Plus, Molecular Devices). Protein for each sample, 60 µg, was loaded to perform electrophoresis in a precast 4–20% SDS-PAGE (Ready Gel Tris-HCl Gel, BioRad) and transferred onto a nitrocellulose membrane (Whatman) using a semi-dry method. Proteins were detected using the following primary antibodies: mouse monoclonal anti-alpha cardiac actin (Sigma, A9357, 1∶1,000), rabbit anti-cardiac troponin I–C (Santa Cruz, sc-98830, 1∶1,000), and rabbit anti-glyceraldehyde-3-phosphate dehydrogenase (Millipore, MAB374, 1∶850). Incubations were performed overnight at 4°C. The secondary antibodies used were anti-Rabbit IgG, horseradish peroxidase-linked whole Ab (GE Healthcare, NA934, 1∶10,000) and anti-mouse IgG, horseradish peroxidase-linked whole Ab (GE Healthcare, NA931, 1∶10,000).

### Statistical analysis

hEB cross sectional area data were analyzed using linear regression, with ROCKi, centrifugation (spin), and day as independent categorical variables (SAS v. 9.3, SAS Institute). Two-way interactions were included. Model assumptions were checked graphically and were met for the BG01V/hOG and the H9 hEB data. Least squares means, which control for other model variables, were calculated. Mean differences between treatments were examined using Tukey's adjustment for multiple comparisons to control type I error rate. The significance level was set at α = 0.05.

## Results

### hESC seeding density for hEB formation

hEB formation from dissociated hESC suspensions was tested using non-adhesive hydrogel microwell arrays. The effect of hESC seeding density on hEB formation was examined. The hESCs were suspended in the culture medium, and the molds were seeded over the range of 10,000 to 35,000 hESCs per microwell in increments of 5,000 hESCs. Formation of hEBs was tested under a total of four treatment conditions (ROCKi: ROCK inhibitor; Spin: centrifugation): -ROCKi/-spin, -ROCKi/+spin, +ROCKi/-spin, and +ROCKi/+spin. The hEBs were transferred from the molds to suspension culture for growth and differentiation assays after 24 hrs of incubation.

The minimum input cell number per microwell required to form a hEB that is able to maintain structural integrity in suspension culture was approximately 10,000 cells per microwell for the BG01V/hOG hESC line, and 15,000 cells per microwell for the H9 line. For each hESC line, there was a range of input cell number (10,000 to 25,000 hESCs per microwell for the BG01V/hOG line, and 15,000 to 30,000 hESCs per microwell for the H9 line) that allowed hEB formation in the agarose hydrogel microwell molds. Input cell numbers that fell off the lower end of the range resulted in disintegration of hEBs within the first two to three days in suspension culture. hESCs failed to aggregate when input cell numbers exceeded the higher end of the range regardless of the presence of ROCKi and/or centrifugation. For all the subsequent investigations, 15,000 hESCs/microwell for the BG01V/hOG cells, and 25,000 hESCs/microwell for the H9 cells were seeded. For each experiment, the same cell suspension was used to form hEBs for each of the four conditions for each cell line.

### Neither ROCKi nor centrifugation was required for hEB formation for both hESC cell lines

**Figure 1:**
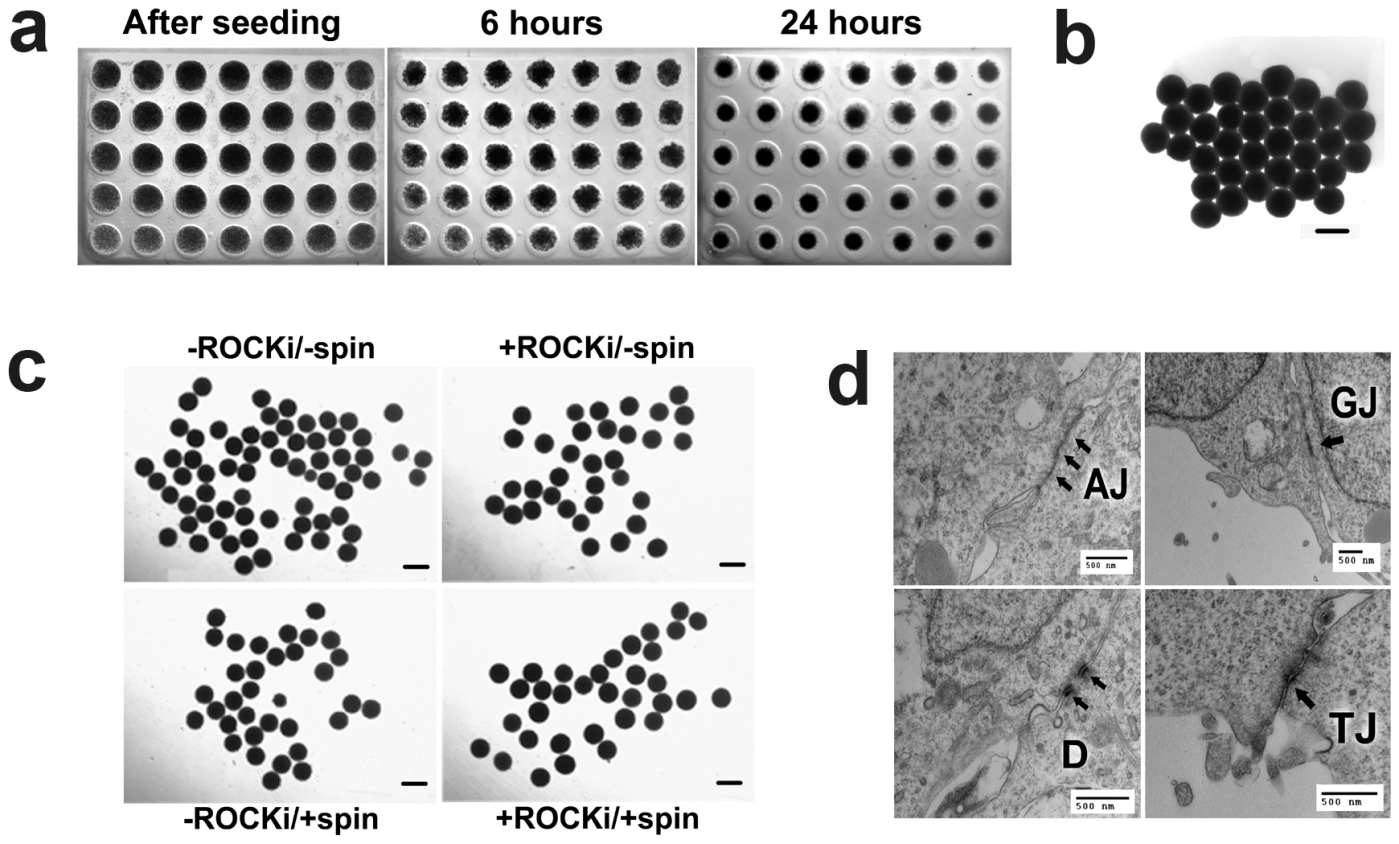
BG01V/hOG cells formed hEBs in the microwells at a cell seeding density of 15,000 hESCs per microwell. (**a**) Condensation of the hESC suspension within the microwells was progressive and evident after 6 hrs of incubation. (**b**) hEBs were compact and spherical and be able to be extracted intact from the microwells after 24 hrs of incubation. (**c**) The freshly extracted hEBs formed under four different conditions (ROCKi: ROCK inhibitor; Spin: centrifugation). (**d**) Internal structural organization among the cells within the freshly extracted hEBs (formed under -ROCK/-spin condition) was demonstrated by the presences of cell-cell junctions, i.e., adherence junctions (AJ), gap junctions (GJ), desmosomes (D), and tight junctions (TJ), in TEM images. All treatments were performed with aliquots from the same cell suspension. Microwell diameter 820 µm. Scale bars 500 µm.

For the BG01V/hOG cells, under each of the four conditions, condensation of the BG01V/hOG cell suspension was visible after 6 hrs of incubation in the microwell arrays (**Fig. 1a**). The cells became more densely aggregated and compact over time in incubation. After 24 hrs of incubation, hEBs could be readily transfer to other cell cultureware (**Fig. 1b**). Notably, neither ROCKi nor centrifugation was required for hEB formation. The formed hEBs were uniform in sizes (Table 1) and nearly spherical for each treatment condition (**Fig. 1c**). Freshly formed hEBs after 24 hrs of incubation displayed a variety of cell-cell junctions, reflecting a complex and increasingly organized internal structure (**Fig.** **1d**).

**Table 1:** Cross-sectional areas (µm^2^) of the hEBs formed after 24 hrs of incubation.

	-ROCKi/-spin	-ROCKi/+spin	+ROCKi/-spin	+ROCKi/+spin
BG01V/hOG	500591 (±26830)	406089 (±17808)	628116 (±23271)	684089 (±19302)
H9	154126 (±6759)	156055 (±7104)	183059 (±5553)	138410 (±9850)

**Figure 2:**
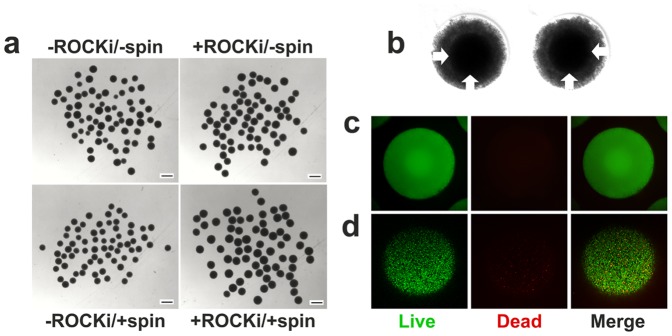
H9 hESCs formed hEBs in the microwells at a cell seeding density of 25,000 hESCs per microwell. (**a**) Freshly extracted hEBs formed under four different conditions in microwells after 24 hrs of incubation. (**b**) A closer look at hEBs formed under -ROCK/-spin condition after 24 hrs of incubation before extraction indicated the presences of a compact core (arrows) and a loosely-aggregated corona. (**c**) Live-dead staining of the formed hEBs under -ROCK/-spin condition after 24 hrs of incubation before transfer to suspension culture indicated high viability (>90%) of hESCs both at the core and the corona (SYTO 10 green-fluorescent nucleic acid stain for all cells; DEAD Red (ethidium homodimer-2) nucleic acid stain for dead cells). (**d**) Confocal microscopic images of live/dead staining of freshly extracted hEBs (formed under -ROCK/-spin condition) indicated high viability (>85%) of the hESCs. Note that cells at the corona of the hEBs were sloughed off during the transfer process. Scale bars 500 µm.

In concurrence, treatment with ROCKi and/or centrifugation was not required for hEB formation by the H9 cells either. Condensation of the hESC suspension within the microwells was also progressive for the H9 cells. However, condensation of the suspension occurred somewhat differently for the two hESC lines. For the BG01V/hOG hESCs, nearly all cells in the microwell condensed to participate in hEB formation. For H9 hESCs, a portion of the cells did not condense, instead, they remained loosely aggregated at the peripheral of the hEBs (**Fig. 2b**). At 24 hrs of incubation, hEBs were readily formed under each of the four conditions (**Fig. 2a**). The formed hEBs were uniform in sizes and spherical (Table 1 and **Fig. 2a**). In particular, regardless of the condition, the hEBs were consisted of a central compact core (**Fig.** **2b**, arrows, showing formed hEBs under -ROCKi/-spin condition as an example) that was surrounded by a loosely-aggregated corona. hESCs at the core and within the corona exhibited a high degree of viability (>90%, **Fig. 2c**). During the transfer process from the microwells to the subsequent suspension culture, cells at the corona were sloughed off. A closer examination of the cells in the extracted hEBs indicated a high degree of viability (>85%, **Fig. 2d**), suggesting the hESCs remain viable under the all four conditions regardless of whether they participate in the hEB formation, and that the transfer process poses little interference to cell viability. Taken together, the non-adhesive hydrogel microwell arrays promoted the formation of uniform-sized, spherical hEBs from both BG01V/hOG and H9 hESCs in the absence of either ROCKi or centrifugation.

### ROCKi and/or centrifugation was associated with hEB size differences for both hESC cell lines

**Figure 3:**
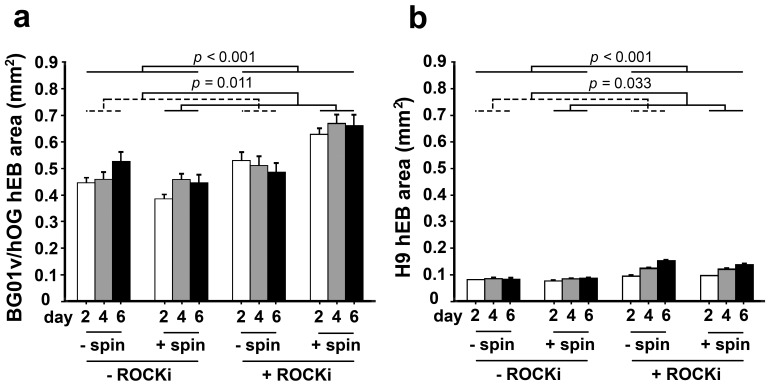
Sizes (the cross-sectional areas, mean ± s.e.m.) over time in suspension culture of hEBs formed under the four different conditions for the (a) BG01V/hOG and (b) H9 cells.

Next, we evaluated whether ROCKi and/or centrifugation treatment during hEB formation would result in differences in hEB sizes. The sizes (cross-sectional areas) of the hEBs formed under different treatment conditions were measured over time at 2, 4, and 6 days in suspension culture for the two hESC cell lines. For the BG01V/hOG cells, treatment with ROCKi during hEB formation was associated with greater hEB cross-sectional area than those –ROCKi groups when controlling for centrifugation and time (*p*<0.001) (**Fig. 3a**). Treatment with centrifugation was also associated with greater hEB cross-sectional area than those no-centrifugation (–spin) groups when controlling for ROCKi and time (*p* = 0.011). There was a significant synergistic effect of ROCKi and centrifugation treatment on increasing the sizes of resultant hEBs. hESCs treated with both ROCKi and centrifugation (+ROCKi/+spin group) had a greater cross-sectional area than hESCs treated with ROCKi (*p*<0.001) or centrifugation alone (*p*<0.001) or untreated hESCs (-ROCKi/-spin group, *p*<0.001) (detailed model results in **Table** S**1**). In all the four treatment groups, the hEB cross-sectional area did not vary over time in suspension culture.

For the H9 cells, treatment with ROCKi during hEB formation was associated with greater area than those –ROCKi groups when controlling for centrifugation and time (*p*<0.001) (**Fig. 3b**). Treatment with centrifugation was associated with a smaller cross-sectional area than those –spin groups when controlling ROCKi and time (*p* = 0.033). There was a significant interaction between ROCKi and centrifugation treatment (*p* = 0.042). H9 hESC treated with ROCKi alone had a greater area than hESC treated with both ROCKi and centrifugation (+ROCKi/+spin group, *p* = 0.017), centrifugation alone (*p*<0.001), or untreated hESCs (-ROCKi/-spin group, *p*<0.001) (detailed model results in **Table** S**2**). There was a significant association between cross-sectional area and day, with area increasing over time for all the four groups (*p*<0.001 for all time point comparisons). There was also a significant interaction between ROCKi treatment and day (*p*<0.001) with a greater increase in area over time in the ROCKi treated hEBs. No significant association was found between centrifugation and culture day.

### hEBs formed under the -ROCKi/-spin condition expressed proteins representative of all the three developmental germ layers

**Figure 4:**
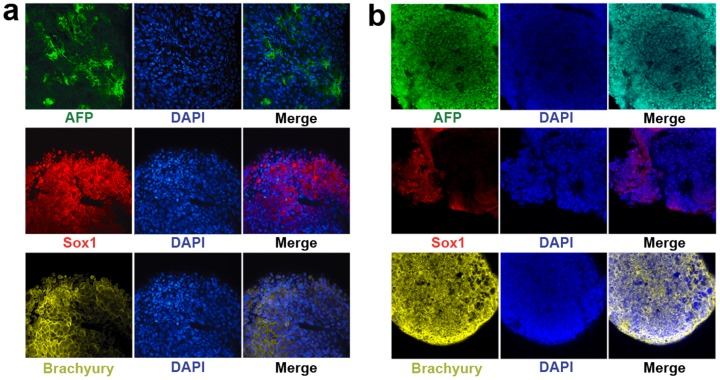
hEBs (formed under -ROCK/-spin condition) expressed proteins characteristic of all the three developmental germ layers. At 20 days in suspension culture, the hEBs were positive for Alpha Fetoprotein (AFP, endoderm-specific), SOX1 (ectoderm-specific), and brachiury (mesoderm-specific) for both the (**a**) BG01V/hOG and (**b**) H9 cell lines. Cell nuclei were stained in blue with DAPI.

Once we demonstrated uniform-sized spherical hEBs were able to form in our hydrogel microwells under the -ROCKi/-spin condition, all subsequent investigations were confined to the hEBs formed under this condition. To confirm the pluripotency of the formed hEBs, it is important to determine whether the hEBs are able to generate representatives of the three developmental germ layers. Immunofluorescent staining of the hEBs (formed from the BG01V/hOG and the H9 hESC lines) at 20 days in suspension culture revealed robust expression of proteins characteristic of all the three developmental germ layers, including Alpha Fetoprotein (AFP, endoderm-specific), SOX1 (ectoderm-specific), and Brachiury (mesoderm-specific) (**Fig. 4a,b**). These data confirm that the formed hESC aggregates are indeed hEBs since they possess the fundamental properties of embryoid bodies by giving rise to all the three developmental germ layers within a timeframe that is comparable to that of in vivo embryogenesis (i.e., in vivo gastrulation ends followed by the formation of the three developmental germ layers at around 20 days after fertilization), and that they are pluripotent, having the potential to differentiate into a full spectrum of lineages associated with each germ layer. The EB stage represents the onset of hESC differentiation.

### hEBs formed under the -ROCKi/-spin condition differentiated into tissue lineages specific to each germ layer

**Figure 5:**
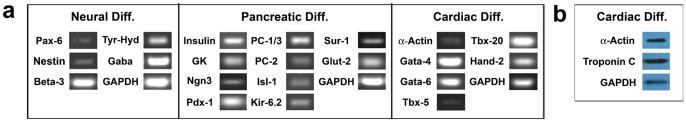
BG01V/hOG hEBs (formed under -ROCK/-spin condition) differentiated into tissue lineages specific to each of the three embryonic germ layers. (**a**) RT-PCR analysis demonstrated gene expression profiles that are specific to representative lineages associated with each germ layer, i.e., neural (ectoderm), pancreatic (endoderm), and cardiac (mesoderm) lineages. (**b**) At 25 days when treated with the cardiac differentiation protocol, western blot confirmed expressions of cardiac α-actin and cardiac troponin-C proteins that are representative of cardiac lineage.

To further confirm that the hEBs formed under the -ROCKi/-spin condition are able to differentiate into tissue lineages specific to each of the three embryonic germ layers, we applied standard differentiation protocols to induce their differentiation towards neural (ectoderm-specific), cardiac (mesoderm-specific), and pancreatic (endoderm-specific) lineages, respectively. We first tested the hEBs derived from the BG01V/hOG cells under the -ROCKi/-spin condition. RT-PCR examination of the cells derived from these hEBs after respective differentiation protocol demonstrated unambiguous expression of a complete panel of lineage-specific genes for each germ layer-specific lineage (**Fig. 5a**). Genes that are characteristic of neural lineage include neuroectodermal markers expressed during neural plate and neural tube-like structure (NT) formation such as Pax-6 (a stage-specific markers for neuro-ectodermal differentiation [30]) and Nestin (an intermediate filament protein expressed in dividing cells during the early stages of development in the central nervous system (CNS) [31] and the peripheral nervous system (PNS) [32], [33]), and markers for mature neural lineages associated with more differentiated neuronal stage such as Beta-3 (β-III-tubulin) (an adrenergic receptor [34]), TYR-HYD (Tyrosin Hydroxylase, an enzyme responsible for catalyzing the conversion of the amino acid L-tyrosine to dihydroxyphenylalanine (DOPA)[35]), and GABA (γ-Aminobutyric acid, the chief inhibitory neurotransmitter in the mammalian CNS that regulates neuronal excitability [36], [37]). Expression of the genes associated with more mature neuronal lineages (such as Beta-3, TYR-HYD, and GABA) at much higher levels when compared to those for early neural markers (such as Pax-6 and Nestin) could indicate that the cells derived from the BG01V/hOG hEBs after neural differentiation are committed to a neural fate rather than residing at a neuroepithelial or neural progenitor stage.

For the cardiac differentiation, the cells expressed genes that are critical to either initiate cardiac specification and/or maintain cardiac phenotype. These include cardiac α-actin, a major constituent of the contractile apparatus that is present in muscle tissues [38]; HAND2, which is asymmetrically expressed in the developing ventricular chambers and plays an essential role in cardiac morphogenesis [39]; TBX5, encodes a protein that plays a role in heart development [40]; TBX20 that is essential for early heart development [41]; GATA4, a protein that regulates genes involved in embryogenesis and myocardial differentiation/function [42], [43]; and GATA6 that is associated with embryonic cardiac development [43]. The higher expression levels of the molecular markers for early cardiac development and morphogenesis (e.g., GATA4, GATA6, TBX20, and HAND2) when compared to those of more chamber-specific cardiac markers (e.g., α-actin and TBX5) may suggest the output cells after cardiac differentiation of the hEBs that were produced under the -ROCKi/-spin condition retained the potential to differentiate into a wide range of cardiac precursors. Cardiac differentiation was also demonstrated by the presence of the cardiac α-actin and troponin proteins detected by western blot (**Fig. 5b**).

Gene expressions were consistent with pancreatic lineage after pancreatic (endodermal) differentiation of the BG01V/hOG cells (**Fig. 5a**). Two sets of pancreatic β-cell-specific genes that either encode proteins of the characteristic structural components of pancreatic β-cells or are the key to de novo synthesis of insulin were detected. Genes in the first category include Ngn3, a basic helix-loop-helix transcription factor that is critical for the development of the endocrine cells of the islets [44]; Glut-2, an integral plasma membrane glycoprotein of islet beta cells which has been suggested as a glucose sensor [45]; Glucokinase (GK), an enzyme that plays an important role in the regulation of carbohydrate metabolism as a glucose sensor by facilitating phosphorylation of glucose to glucose-6-phosphate [46]; and Kir-6.2, pancreatic β-cell-expressing adenosine triphosphate (ATP)-sensitive potassium (K_ATP_) channels that are needed for normal insulin secretion [47]. Genes pertinent to insulin secretion include PDX-1 (also known as Insulin Promoter Factor 1), a transcriptional activator of several genes necessary for pancreatic development, β-cell maturation, and insulin secretion [48]; PC-1/3 and PC-2, two key enzymes (endopeptidases or prohormone convertases) possessing a proinsulin-processing endopeptidase activity through cleavage of specific sites on proinsulin to convert to insulin [49]; Sur-1, a protein that modulates K_ATP_ channels and insulin release [50]; and Islet-1, a protein that binds an enhancer region of the insulin gene, among others, which may play an important role in regulating insulin gene expression [51]. The presence of insulin was also clear (**Fig. 5a**).

**Figure 6:**
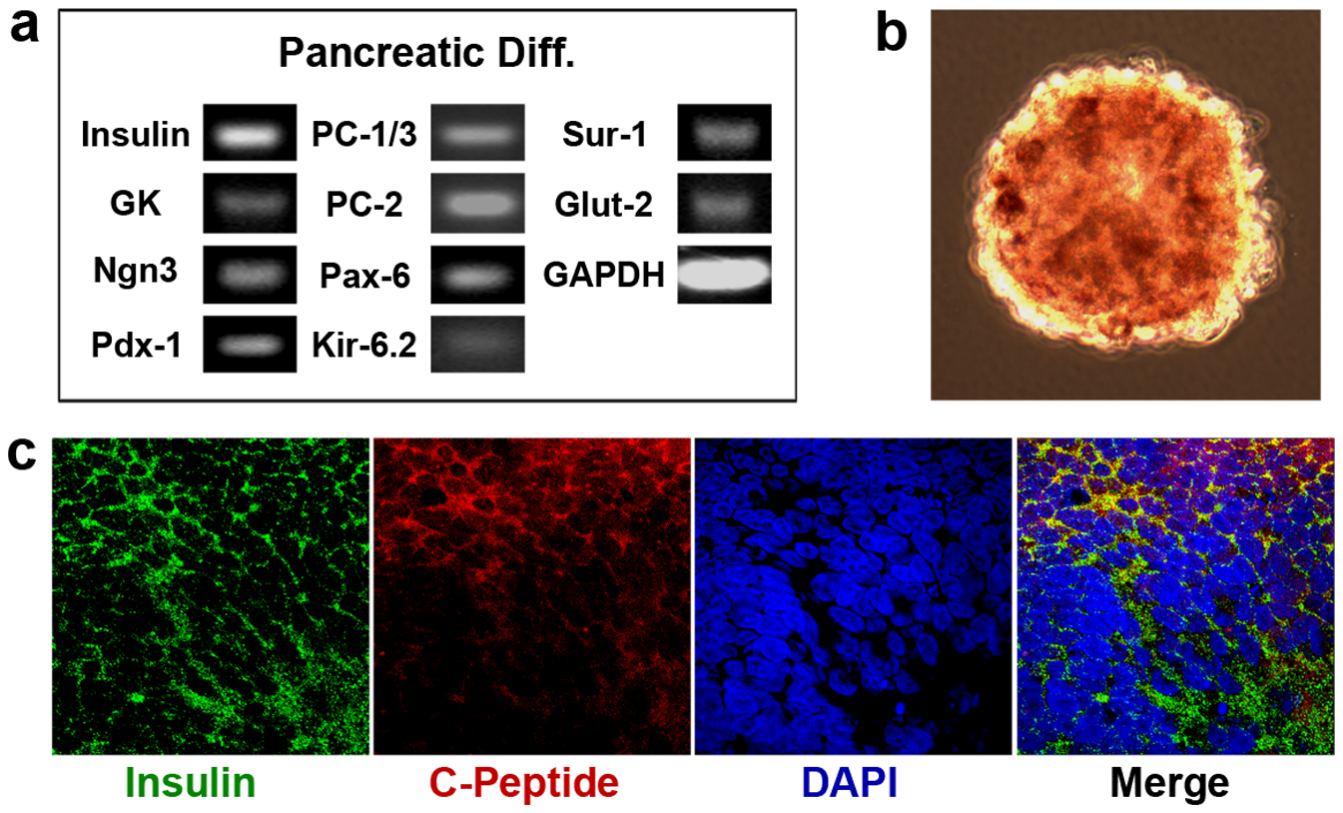
H9 hEBs (formed under -ROCK/-spin condition) differentiated into pancreatic lineage at 18 days when treated with the pancreatic differentiation protocol. (**a**) RT-PCR analysis demonstrated expression of key pancreatic lineage-specific genes. (**b**) Over 90% of the cells were insulin-positive, as evidenced by positive staining for dithizone (DTZ) in dark red. (**c**) Immunostaining of the cells revealed co-localization (in yellow) of insulin and C-peptide. Cell nuclei were stained in blue with DAPI.

In parallel, gene expression was characteristic of pancreatic lineage after pancreatic differentiation of the H9 hEBs. After 21 days of pancreatic differentiation with our protocol, over 90% of the cells were insulin-positive, as evidenced by the positive staining for Dithizone (DTZ) (**Fig. 6b** DTZ+ in dark red color), a specific enzyme that indicates the presence of insulin [52]. RT-PCR analysis of the cells revealed expressions of key pancreatic β-cell-specific genes, including insulin, Pdx-1, PC-1/3 and PC-2, GK, Ngn3, Pax-6, Sur-1, Glut-2, and Kir-6.2 (**Fig. 6a**). Immunofluorescence double staining of insulin and C-peptide revealed their co-localization on the insulin-producing cells (**Fig. 6c**), confirming a de novo synthesis of insulin that is indicative of the full functionality of our differentiated cells. C-peptide is a by-product when proinsulin is converted to insulin. In all, after pancreatic differentiation, the H9 EBs that were produced under the -ROCKi/-spin condition were able to give rise to mature functional insulin-producing pancreatic β-cells, an endodermal-specific lineage.

## Discusssion

In this study, we have developed a simple technology based upon non-adhesive hydrogel microwell arrays for the formation of hEBs from dissociated single cell suspension of hESCs. We have, for the first time, demonstrated robust hEB formation from singularized hESCs in the absence of ROCKi or centrifugation. For both hESC cell lines that were tested, i.e., BG01V/hOG and H9, within a range of cell seeding density per microwell, uniform-sized spherical hEBs were formed under no-ROCKi and no-spin condition, and extracted intact from the microwells for subsequent culture/differentiation. The hEBs exhibited organized internal tissue-level structures and expressed proteins distinctive for all three embryonic germ layers. Exposure of the hEBs to directed differentiation protocol led to their specifications into lineages characteristic of each germ layer.

Dissociated single cell suspension represents a more uniform inputting population for hEB formation when compared to cell colonies/clumps that are widely variable in terms of sizes and pre-existing organizations [53]. Single cell suspension also allows strict control of EB sizes by seeding defined numbers of cells per microwell for the formation of one discrete EB per well. However, when present in the form of dissociated single cell suspension, hESCs have displayed extremely low viability [54], [55], possibly due to the inability to establish cell-cell contact. hESCs cultured in 3-D microwell arrays have demonstrated higher expression of E-cadherins than cells on 2-D substrates. In addition, hEBs formed from colonies of hESCs cultured in 3-D microwells displayed much higher levels of Wnt signaling than hEBs formed from hESCs in 2-D cultures [56]. Both E-cadherins [26] and Wnt signaling are implicated in cell junctions. These results suggest that 3-D microwells facilitate cell-cell contact in hESCs. The study that led to direct correlation between cell-cell contact and cell viability was performed on cells other than hESCs, e.g., pancreatic beta-cells, which exhibited multi-fold increase in viability in the presence of cell-cell contact formed during aggregation into clusters in 3-D microwells when compared to singularized cells of the same type [57]. Therefore, one challenge to be addressed in any techniques for effective hEB formation starting from dissociated hESCs is to allow quick and controlled aggregation to minimize the residential time of hESCs in the form of dissociated single cells, and increase cell-cell interactions.

To this end, we have developed deep round-bottom non-adhesive hydrogel microwell arrays for hEB formation. Non-adhesive or low-adherence microwells have been previously used in the attempts of other labs to generate hEBs with variable results. Some studies have shown moderate success, e.g., hEBs were able to form in microwells after forced aggregation via centrifugation (“the spin EBs”), but the survival and differentiation remained unpredictable [28]. Others have shown disintegration of the hESC cell pellets (formed without centrifugation) into undefined variable 3-D structures that were difficult to be extracted from the microwells [15]. Centrifugation has been shown to cause mechanical injury to cells, destruct cell membranes, and may alter the stem characteristics of stem cells and their differentiation potential [58]. It also prevents scale-up and automation of the process due to manual procedure and intense labor in sample handling. Recently, ROCKi Y-27632 has been adapted to enhance the survival of singularized hESCs in culture while preserving their pluripotency [19]. In the presence of ROCKi, but the absence of centrifugation, coherent hEBs were able to form from dissociated hESCs that were cultured on the Matrigel, indicating the effectiveness of ROCKi on hEB formation [15]. The survival-promoting effect of ROCKi on dissociated hESCs was most prominent during the initial stage following the dissociation/replating procedure [19], which represents a critical time period for cell survival. The underlying mechanism responsible for the observed effect of ROCKi is not known, yet it may be related to its ability to amplify anti-apoptotic signals upon cellular detachment and dissociation [26], [59], likely in an anti-apoptotic manner that is similar to the survival-promoting effect of neurotrophins on hESCs [55]. Several studies have demonstrated successes in homogenous and synchronized hEB formation from singularized hESCs in the presence of ROCKi [19]. However, ROCKi is a xenofactor, and even short-term exposure of hESCs to ROCKi has been shown to bias cell fate toward residual pluripotency, rendering the cells unsuitable for patient therapies [8]. To date, the present study is the first report on successful generation of uniform-sized synchronized hEBs from singularized hESCs in the absence of centrifugation or ROCKi. The fabricated deep round-bottom microwell arrays of agarose, a neutral hydrophilic polymer that is non-adherent to cells, had allowed quick robust aggregation among the singularized hESCs for hEB formation without the need for centrifugation or ROCKi.

Successful hEB formation occurred only within a specific range of input hESC cell number per microwell, which was similar for both hESC lines. Use of a higher or a lower number of cells outside of the range resulted in the failure of the hESCs to aggregate, or the formation of loose aggregates that disintegrated immediately when transferred to suspension culture. This cell number dependence was a feature of both hESC lines whether or not the cells were treated with ROCKi or centrifugation. Our experiments used a deep round-bottom microwell of a single size; additional testing will be required to determine if the cell number range for successful hEB formation varies with well size or geometry. Treatment of the hESCs with ROCKi and/or centrifugation altered hEB sizes. ROCKi was associated with larger hEBs for both cell lines. Centrifugation was associated with larger hEBs for the BG01V/hOG line and smaller hEBs for the H9 line. These size differences are evidence of the immediate effects of ROCKi and/or centrifugation treatments on the resultant hEBs. Our hEB formation strategy eliminates the need for these treatments and any down-stream effects that they may have.

Further demonstration of the ability of the hEBs to generate all three embryonic germ layers confirmed the pluripotency of the hEBs. When exposed to specific lineage differentiation protocols, the hEBs differentiated into each of the embryonic cell lineages. These results have suggested that our microwell array-based hEB formation strategy under the no-ROCKi no-spin condition has indeed produced hEBs rather than hESC aggregates or colonies, and it does not interfere with the differentiation potential or pluripotency of the hEBs to deliver a full spectrum of clinically-relevant lineages for cell therapies. The findings have also justified the use of the formed hEBs as a model system that recapitulates the early stages of development for in vitro organogenesis and research on human developmental biology.

In addition to maintaining high viability of hESCs during hEB formation, the other challenge is to improve the efficiency, homogeneity, and reproducibility of the process [3], [22]. Efficiency is important to achieve high through-put, automated, and scalable production of hEBs for research and clinical interests. To date, the major barrier to improve efficiency is the centrifugation step for forced aggregation. By eliminating this step, this non-adhesive microwell array-based approach represents a trend in high through-put technology for process development. 80% confluent hESCs cultured in a 6-well plate may provide sufficient numbers of cells to seed 280 microwells of 820 µm in diameter, therefore, allowing production of 280 hEBs at the same time. General cell replacement therapy requires nearly 10^8^∼10^9^ cells per patient per administration [60]. Given the number of cells contained in each hEB, the yield of directed differentiation, and the viability and functionality of the transplanted cells, each administration per patient requires approximately 50∼70 6-well plates of hESC culture for hEB preparation using our microwell arrays. The labor- and space-efficient, and scalable technology that we have developed in the present study for hEB production in large quantities would fuse into the mainstream of research efforts in our lab on establishing fully automatic robotic platform for the culture and directed differentiation of human pluripotent stem cells, such as hESCs and hiPSCs (human induced pluripotent stem cells). Thousands of the microwell arrays can be fitted into small space and automatically processed in the same way without the need for ROCKi or centrifugation, thus ensuring the homogenecity and synchronization of the resultant hEBs as opposed to heterogeneous and unsynchronized output populations that are associated with conventional hEB formation protocols [21], [61]. The produced hEBs in large batches may be further induced to generate large numbers of differentiated cells for clinical-applicable cell therapies.

Throughout the study, there are several notable differences between the two hESC cell lines, i.e., BG01V/hOG and H9. In the BG01V/hOG cells, nearly all of the hESCs in each microwell condensed to form a robust hEB. In the H9 cells, the cells remained viable in culture in the microwells; however, only a portion of the cells in each microwell formed a dense core while the remaining cells formed a loose halo of the aggregate, which was then sloughed off during transfer. As a result, under the same treatment condition, hEBs formed from H9 line seemed to be less compact. Due to the fact that both hESC cell lines underwent the same treatment for hEB formation, the observed differences are cell line-dependent rather than technology-dependent. Differences in hEB formation among hESC lines have been reported previously [27]. Parallel interline comparison of several hESC lines in terms of hEB formation (from dissociated single cells with centrifugation), growth, and cardiomyocyte differentiation implicated the derivation and culture history of each line as a source of variability [27]. Since BG01V/hOG and H9 are independently derived hESC lines [62], [63], the observed differences may be linked to both inherent genetic differences and epigenetic differences [27], even though differences in input cell density and the culture medium may also play roles [64]. Additional testing will be required to determine the generic interline utility of this deep round-bottom microwell-based strategy in hEB formation among other independently-derived hESC lines without ROCKi or centrifugation treatment.

## Conclusion

Taken together, we have demonstrated the utility of non-adhesive agarose hydrogel microwell arrays for the formation of hEBs from dissociated single cell suspension of hESCs in the absence of ROCKi or xeno factors or the rate-limiting step of centrifugation – in a scalable and cost-effective manner. The formed hEBs were homogeneous in size and shape without pre-existing cell-cell contacts, and developed the three embryonic germ layers and tissues derived from each of the germ layers. Our strategy will directly support automated, large-scale production of hEBs or hESC-derived cells required for clinical, research, or therapeutic applications.

## Supporting Information

Figure S1 **(A) Image of a Teflon stamp with micronipple arrays that was used to create the hydrogel microwells for the hEB formation; (B) Low-melting-point agarose was used to make microwells to form hEBs.** The agarose was dissolved in 1X phosphate buffered saline (PBS) at 100°C and pipetted into the culture ware. After approximately 10 min, the agarose gelled, the Teflon micronipple stamp was withdrawn, and hydrogel microwells with replicative geometry of the micronipple stamp were formed into the gel; A 50 µl dissociated single-cell suspension of hESCs was dispensed into each mold to evenly fill all the microwells. After 24 hours of incubation, the formed hEBs were taken out from microwells, imaged, and transferred to suspension culture.
(TIF)

Table S1 **LSMeans^a^ and Tukeys post hoc comparisons for the cross sectional area of hEBs formed using approx. 15,000 BG01V/hOG hESC/well.** Twenty-eight to forty-four hEBs were evaluated per group at each time point.
(DOC)

Table S2 **LSMeans^a^ and Tukeys post hoc comparisons for the cross sectional area of hEBs formed using approx. 25,000 H9 hESC/well.** Seventy hEBs were evaluated per group at each time point.
(DOC)
