# Supplementary figures and images for: ROCK Inhibitor Is Not Required for Embryoid Body Formation from Singularized Human Embryonic Stem Cells
Source: PLoS One. 2014 Nov 3;9(11):e100742. doi: 10.1371/journal.pone.0100742 (PMC4217711; doi:10.1371/journal.pone.0100742)

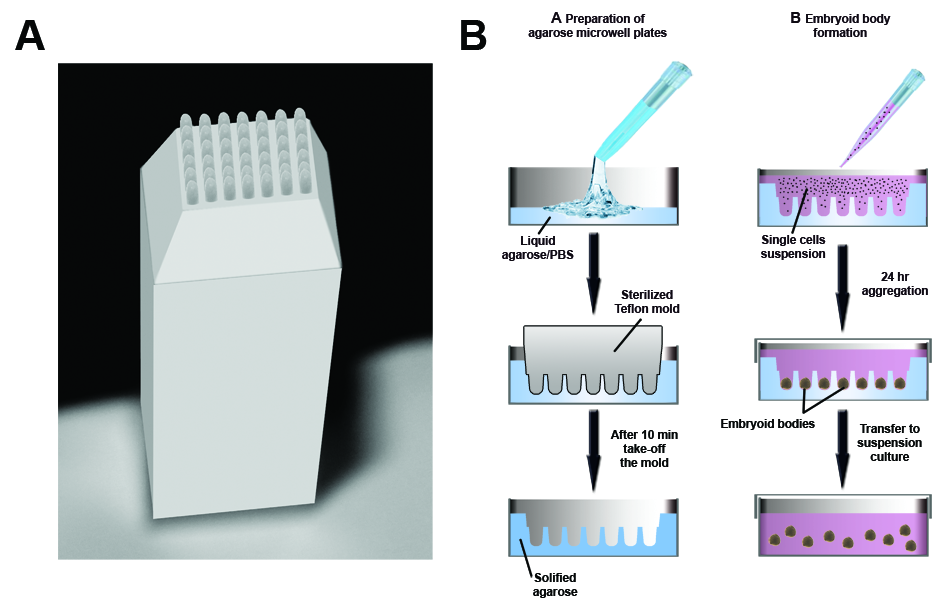

Supplement: Figure S1 — (A) Image of a Teflon stamp with micronipple arrays that was used to create the hydrogel microwells for the hEB formation; (B) Low-melting-point agarose was used to make microwells to form hEBs. The agarose was dissolved in 1X phosphate buffered saline (PBS) at 100°C and pipetted into the culture ware. After approximately 10 min, the agarose gelled, the Teflon micronipple stamp was withdrawn, and hydrogel microwells with replicative geometry of the micronipple stamp were formed into the gel; A 50 µl dissociated single-cell suspension of hESCs was dispensed into each mold to evenly fill all the microwells. After 24 hours of incubation, the formed hEBs were taken out from microwells, imaged, and transferred to suspension culture. (TIF) [file pone.0100742.s001.tif]
