# Supplementary material for: ROCK Inhibitor Is Not Required for Embryoid Body Formation from Singularized Human Embryonic Stem Cells
Source: PLoS One. 2014 Nov 3;9(11):e100742. doi: 10.1371/journal.pone.0100742 (PMC4217711; doi:10.1371/journal.pone.0100742)
Supplement: Table S1 — LSMeansa and Tukeys post hoc comparisons for the cross sectional area of hEBs formed using approx. 15,000 BG01V/hOG hESC/well. Twenty-eight to forty-four hEBs were evaluated per group at each time point. (DOC) [file pone.0100742.s002.doc]

**Table S1:** LSMeans^a^ and Tukeys post hoc comparisons for the cross sectional area of hEBs formed using approx. 15,000 BG01V/hOG hESC/well. Twenty-eight to forty-four hEBs were evaluated per group at each time point.

| Variable | Model  *p*-value | Status | LS Mean^a^ [mm^2^]  Area (95% CI) SE | Tukey post hoc comparison  *p*-value | |
| --- | --- | --- | --- | --- | --- |
| ROCKi^b^ | <0.001 | − | 0.455 (0.427, 0.482) 0.014 | <0.001 |  |
|  |  | + | 0.582 (0.557, 0.607) 0.013 |  |  |
| spin^c^ | 0.011 | − | 0.494 (0.468, 0.520) 0.013 | 0.011 |  |
|  |  | + | 0.542 (0.516, 0.569) 0.014 |  |  |
| day | 0.375 | 2 | 0.499 (0.467, 0.530) 0.016 | 1 v. 2 0.482 |  |
|  |  | 4 | 0.525 (0.493, 0.558) 0.017 | 1 v. 3 0.356 |  |
|  |  | 6 | 0.531 (0.498, 0.563) 0.017 | 2 v. 3 0.973 |  |
| ROCKi * spin | <0.001 | (a) −ROCKi, −spin | 0.478 (0.439, 0.518) 0.020 | a v. b: 0.339 | b v. d: <0.001 |
|  |  | (b) −ROCKi, +spin | 0.431 (0.392, 0.470) 0.020 | a v. c: 0.639 | c v. d: <0.001 |
|  |  | (c) +ROCKi, −spin | 0.510 (0.476, 0.544) 0.017 | a v. d: <0.001 |  |
|  |  | (d) +ROCKi, +spin | 0.654 (0.617, 0.691) 0.019 | b v. c: 0.015 |  |
| ROCKi * day | 0.232 | (a) −ROCKi, day 2 | 0.417 (0.370, 0.464) 0.024 | a v. b: 0.814 | b v. d: 0.004 |
|  |  | (b) −ROCKi, day 4 | 0.460 (0.411, 0.509) 0.025 | a v. c: 0.305 | b v. e: 0.001 |
|  |  | (c) −ROCKi, day 6 | 0.487 (0.439, 0.535) 0.024 | a v. d: <0.001 | b v. f: 0.011 |
|  |  | (d) +ROCKi, day 2 | 0.580 (0.538, 0.623) 0.022 | a v. e: <0.001 | c v. d: 0.052 |
|  |  | (e) +ROCKi, day 4 | 0.591 (0.548, 0.633) 0.022 | a v. f: <0.001 | c v. e: 0.020 |
|  |  | (f) +ROCKi, day 6 | 0.574 (0.529, 0.618) 0.023 | b v. c: 0.969  d v. e: 0.999  d v. f: 1.000 | c v. f: 0.102  e v. f: 0.994 |
| spin * day | 0.458 | (a) −spin, day 2 | 0.489 (0.445, 0.533) 0.022 | a v. b: 1.000 | b v. d: 0.987 |
|  |  | (b) −spin, day 4 | 0.487 (0.442, 0.532) 0.023 | a v. c: 0.995 | b v. e: 0.183 |
|  |  | (c) −spin, day 6 | 0.506 (0.460, 0.552) 0.023 | a v. d: 0.991 | b v. f: 0.305 |
|  |  | (d) +spin, day 2 | 0.508 (0.462, 0.554) 0.023 | a v. e: 0.197 | c v. d: 1.000 |
|  |  | (e) +spin, day 4 | 0.564 (0.517, 0.610) 0.024 | a v. f: 0.321 | c v. e: 0.508 |
|  |  | (f) +spin, day 6 | 0.555 (0.509, 0.602) 0.024 | b v. c: 0.993  d v. e: 0.557  d v. f: 0.720 | c v. f: 0.673  e v. f: 1.000 |

^a^ Least squares mean (all other model variables held constant), ^b^ Rho-associated protein kinase inhibitor, ^c^ Spin denotes centrifugation of the sedimented cell suspension in the microwell microarray.
