# Supplementary material for: ROCK Inhibitor Is Not Required for Embryoid Body Formation from Singularized Human Embryonic Stem Cells
Source: PLoS One. 2014 Nov 3;9(11):e100742. doi: 10.1371/journal.pone.0100742 (PMC4217711; doi:10.1371/journal.pone.0100742)
Supplement: Table S2 — LSMeansa and Tukeys post hoc comparisons for the cross sectional area of hEBs formed using approx. 25,000 H9 hESC/well. Seventy hEBs were evaluated per group at each time point. (DOC) [file pone.0100742.s003.doc]

**Table S2:** LSMeans^a^ and Tukeys post hoc comparisons for the cross sectional area of hEBs formed using approx. 25,000 H9 hESC/well. Seventy hEBs were evaluated per group at each time point.

| Variable | Model  *p*-value | Status | LS Mean^a^ [mm^2^]  Area (95% CI) SE | Tukey post hoc comparison  *p*-value | |
| --- | --- | --- | --- | --- | --- |
| ROCKi^b^ | <0.001 | − | 0.082 (0.080, 0.084) 0.001 | <0.001 |  |
|  |  | + | 0.121 (0.119, 0.123) 0.001 |  |  |
| spin^c^ | 0.033 | − | 0.103 (0.101, 0.105) 0.001 | 0.033 |  |
|  |  | + | 0.100 (0.098, 0.102) 0.001 |  |  |
| day | <0.001 | 2 | 0.087 (0.084, 0.089) 0.001 | 2 v. 4 <0.001 |  |
|  |  | 4 | 0.103 (0.101, 0.105) 0.001 | 2 v. 6 <0.001 |  |
|  |  | 6 | 0.115 (0.112, 0.117) 0.001 | 4 v. 6 <0.001 |  |
| ROCKi * spin | 0.042 | (a) −ROCKi, −spin | 0.082 (0.080, 0.085) 0.001 | a v. b: 0.999 | b v. d: <0.001 |
|  |  | (b) −ROCKi, +spin | 0.082 (0.079, 0.085) 0.001 | a v. c: <0.001 | c v. d: 0.017 |
|  |  | (c) +ROCKi, −spin | 0.124 (0.121, 0.126) 0.001 | a v. d: <0.001 |  |
|  |  | (d) +ROCKi, +spin | 0.118 (0.115, 0.121) 0.001 | b v. c: <0.001 |  |
| ROCKi * day | <0.001 | (a) −ROCKi, day 2 | 0.078 (0.075, 0.081) 0.002 | a v. b: 0.808 | b v. d: <0.001 |
|  |  | (b) −ROCKi, day 4 | 0.084 (0.081, 0.088) 0.002 | a v. c: 0.066 | b v. e: <0.001 |
|  |  | (c) −ROCKi, day 6 | 0.084 (0.081, 0.088) 0.002 | a v. d: <0.001 | b v. f: <0.001 |
|  |  | (d) +ROCKi, day 2 | 0.096 (0.092, 0.099) 0.002 | a v. e: <0.001 | c v. d: <0.001 |
|  |  | (e) +ROCKi, day 4 | 0.122 (0.119, 0.125) 0.002 | a v. f: <0.001 | c v. e: <0.001 |
|  |  | (f) +ROCKi, day 6 | 0.145 (0.142, 0.148) 0.002 | b v. c: 1.000  d v. e: <0.001  d v. f: <0.001 | c v. f: <0.001  e v. f: <0.001 |
| spin * day | 0.296 | (a) −spin, day 2 | 0.087 (0.084, 0.091) 0.002 | a v. b: <0.001 | b v. d: <0.001 |
|  |  | (b) −spin, day 4 | 0.104 (0.101, 0.108) 0.002 | a v. c: <0.001 | b v. e: 0.946 |
|  |  | (c) −spin, day 6 | 0.118 (0.114, 0.121) 0.002 | a v. d: 0.994 | b v. f: 0.022 |
|  |  | (d) +spin, day 2 | 0.086 (0.083, 0.090) 0.002 | a v. e: <0.001 | c v. d: <0.001 |
|  |  | (e) +spin, day 4 | 0.102 (0.099, 0.105) 0.002 | a v. f: <0.001 | c v. e: <0.001 |
|  |  | (f) +spin, day 6 | 0.112 (0.108, 0.115) 0.002 | b v. c: <0.001  d v. e: <0.001  d v. f: <0.001 | c v. f: 0.135  e v. f: <0.001 |

^a^ Least squares mean (all other model variables held constant), ^b^ Rho-associated protein kinase inhibitor, ^c^ Spin denotes centrifugation of the sedimented cell suspension in the microwell microarray.
